# Quantifying magnetic anisotropy using X-ray and neutron diffraction

**DOI:** 10.1107/S2052252521008290

**Published:** 2021-09-01

**Authors:** Emil Andreasen Klahn, Emil Damgaard-Møller, Lennard Krause, Iurii Kibalin, Arsen Gukasov, Shalini Tripathi, Abinash Swain, Maheswaran Shanmugam, Jacob Overgaard

**Affiliations:** a Department of Chemistry, Aarhus University, Langelandsgade 140, Aarhus C 8000, Denmark; b LLB, CEA, CE de Saclay, Gif sur Yvette 91191, France; c Department of Chemistry, IIT Bombay, Powai, Mumbai, Maharashtra 400076, India

**Keywords:** magnetic anisotropy, single-molecule magnets, synchrotron diffraction, polarized neutron diffraction, charge, spin and momentum densities, materials science, magnetic structures

## Abstract

In this work, the magnetic anisotropy in two iso-structural distorted tetrahedral Co(II) complexes, Co*X*_2_tmtu_2_ [*X* = Cl(**1**) and Br(**2**), tmtu = tetra­methyl­thio­urea] is investigated, using a combination of polarized neutron diffraction (PND), very low-temperature high-resolution synchrotron X-ray diffraction and CASSCF/NEVPT2 *ab initio* calculations. Here, it was found consistently among all methods that the compounds have an easy axis of magnetization pointing nearly along the bis­ector of the compression angle, with minute deviations between PND and theory. Importantly, this work represents the first derivation of the atomic susceptibility tensor based on powder PND for a single-molecule magnet and the comparison thereof with *ab initio* calculations and high-resolution X-ray diffraction. Theoretical *ab initio* ligand field theory (AILFT) analysis finds the *d*_*xy*_ orbital to be stabilized relative to the *d*_*xz*_ and *d*_*yz*_ orbitals, thus providing the intuitive explanation for the presence of a negative zero-field splitting parameter, *D*, from coupling and thus mixing of *d*_*xy*_ and . Experimental *d*-orbital populations support this interpretation, showing in addition that the metal–ligand covalency is larger for Br-ligated **2** than for Cl-ligated **1**.

## Abstract 2

A combination of powder and single-crystal polarized neutron diffraction, very low temperature synchrotron X-ray diffraction and *ab initio* calculations can be used to quantify and explain the magnetic anisotropy in Co(II) single-molecule magnets.

## 1. Introduction   

Molecular magnetism (Kahn, 1993 ▸; Gatteschi *et al.*, 2006 ▸) is one particular branch of science where researchers have been able to build a level of understanding that allows the prediction (Rinehart & Long, 2011 ▸; Chilton *et al.*, 2015 ▸) and subsequent realization of a single-molecule magnet with a colossal energy barrier towards magnetic relaxation (Goodwin *et al.*, 2017 ▸; Guo *et al.*, 2018 ▸, 2017 ▸). This was a groundbreaking result for the science of single-molecule magnets (SMMs), as well as a massive boost of faith in traditional scientific behavior.

SMMs are molecules that exhibit a unique magnetic axis, along which an external magnetic field can easily align the molecular magnetization vector either parallel or anti-parallel. We can view this as assuming values of either ‘0’ or ‘1’, in which case the molecule itself becomes a carrier of binary information. Molecules belonging to this class will resist the loss of directionality of the magnetization, and we may consider them as miniature memory bits. Unfortunately, the above-mentioned SMMs are not ideal for building new technology, as they are coordinatively unsaturated lanthanide-ions (and most often dysprosium) which are generally unstable and difficult to handle (Gupta *et al.*, 2016 ▸). Instead, focus could be placed on ions that are more abundant and yield more stable compounds, and there is general consensus in the literature that Co(II) is an ideal target. It exhibits a large spin-orbit coupling parameter; has an odd number of electrons meaning it is a Kramers ion, which significantly reduces the detrimental effects of quantum tunneling that shortcuts the relaxation barrier; and most of its complexes (with coordination number ≥ 4) are stable under ambient conditions.

At the very heart of SMM function is the phenomenon of magnetic anisotropy. This quantifies the difference in the magnetic response when an external magnetic field is applied in different directions relative to the molecule. One caveat is that, although high-level *ab initio* calculations are able to calculate both magnetic anisotropy (Neese *et al.*, 2019 ▸) and, recently, magnetic relaxation properties (Reta *et al.*, 2021 ▸), such calculations are experts tools, and we are still not able to predict the exact magnetic properties of such compounds from a simple set of rules.

The origin of magnetic anisotropy is the presence of orbital angular momentum in the ground state or in a relatively near-excited state. For transition metals, we often quantify this anisotropy using the zero-field splitting parameter (*D*). We are able to estimate the value of *D* from high-level theory (Chibotaru, 2015 ▸), which provides a magnitude and a direction of the anisotropy and also allows an explanation that is based on the electronic structure (Gomez-Coca *et al.*, 2013 ▸). Energy differences between states and the relative magnitude of the eigenvectors of the *g*-tensor can also be experimentally measured using spectroscopic methods such as EPR and INS (Abragam & Bleaney, 2012 ▸; Sigrist *et al.*, 2015 ▸), but these approaches provide no geometrical insight into the direction of the anisotropy axes relative to the molecular structure. Thus, the only link that binds theory and experiment together is the comparison of the *D* values, which in itself is of very limited use as a design criterion. Therefore, to reach a quantitative correlation, we need to measure direction-dependent properties, and this is exactly the focus of this work.

It has been shown on several occasions that four-coordinate Co(II) complexes in distorted tetrahedral geometries exhibit very high negative *D*-values, and some explanations have been presented by us and others (Zadrozny *et al.*, 2013 ▸; Zadrozny & Long, 2011 ▸; Tripathi *et al.*, 2019 ▸; Vaidya *et al.*, 2018 ▸, 2016 ▸, 2014 ▸; Rechkemmer *et al.*, 2016 ▸; Damgaard-Møller *et al.*, 2020*c* ▸). In the current work, we combine results from neutron and synchrotron diffraction to provide irrefutable experimental evidence for the origin of this behavior. Thus, we have re-examined the previously published Co(II)-complexes, Co*X*_2_tmtu_2_ [tmtu = tetra­methyl­thio­urea, *X* = Cl (**1**), Br (**2**)]. Detailed magnetic data of these two complexes were reported by some of us (Vaidya *et al.*, 2018 ▸) and the dc magnetic data presented therein is recalled here for comparison. The temperature-dependent powder magnetic susceptibility data [χ_*M*_ *T*(*T*)] in the temperature range 2–300 K are shown in Fig. S1. The χ_*M*_ *T* value of both complexes **1** and **2** decreases gradually from room temperature to 40 K, below this temperature χ_*M*_ *T* drops precipitously and reaches a final value of 0.8 and 1.2 cm^3^ K mol^−1^ at 2.0 K, respectively. The low temperature drop in χ_*M*_ *T* in both complexes was attributed to the magnetic anisotropy associated with the *S* = 3/2 ground state of **1** and **2**. Consistent with this observation, the magnetic moment of both **1** and **2** does not show any sign of magnetization saturation, even at 70 kOe external magnetic field at 2.0 K in the field-dependent magnetization measurements. Significantly low magnetic moment values for **1** (2.07 *N*μ_B_) and **2** (2.13 *N*μ_B_) at this limit (70 kOe and 2.0 K) further strongly corroborate the presence of relatively large magnetic anisotropy in these structurally analogous complexes. To quantify the spin Hamiltonian (SH) parameters associated with these complexes (both **1** and **2**), the magnetic data [χ_*M*_(*T*) and *M*(*H*)] were fitted simultaneously providing the following SH parameters for **1**, *D* = −18.1 cm^−1^, *g*_iso_ = 2.26; and for **2**, *D* = −16.4 cm^−1^, *g*_iso_ = 2.33. We emphasize the good agreement between the experimental and simulated magnetic data [χ_*M*_ *T*(*T*)] using the computed SH parameters (*g_x_* = 2.25, *g_y_* = 2.28 and *g_z_* = 2.48, *D* = −18.57 cm^−1^. ∣*E*/*D*∣ = 0.067 for **1**; and *g_x_* = 2.26, *g_y_* = 2.30 and *g_z_* = 2.49, *D* = −17.75 cm^−1^, ∣*E*/*D*∣ = 0.086 for **2**). This shows that the incorporation of a transverse component in the simulation of magnetic data does not significantly alter the magnetic anisotropy (*D*) extracted experimentally in the previous study (Vaidya *et al.*, 2018 ▸), where values of −18.1 cm^−1^ and −16.4 cm^−1^ for **1** and **2** were obtained using *g*_iso_ and axial anisotropy. This is presumably due to the relatively small ∣*E*/*D*∣ value associated with these complexes.

Recently, some of us have shown how polarized neutron diffraction (PND) from single crystals can provide accurate magnetic susceptibility tensors, clearly showing the direction and size of the easy axis of magnetization (Klahn *et al.*, 2018 ▸; Ridier *et al.*, 2016 ▸; Gukasov & Brown, 2002 ▸; Tripathi *et al.*, 2021 ▸). Now, a potentially disruptive innovation allows similar information to be obtained from PND on powder (pPND) samples (Kibalin & Gukasov, 2019 ▸) and we present the first thorough analysis using this approach. In a parallel research path, we have recently shown how the *d*-orbital populations obtained from multipole modeling of single-crystal X-ray diffraction (Holladay *et al.*, 1983 ▸) provide significant insight into the magnetic properties of transition-metal based SMMs (Craven *et al.*, 2018 ▸; Thomsen *et al.*, 2019 ▸; Bunting *et al.*, 2018 ▸; Damgaard-Møller *et al.*, 2020*a* ▸,*b* ▸,*c* ▸). In the current work, we have, for the first time, combined these advanced diffraction techniques to obtain both the electronic and the magnetic structure, and added theoretical calculations, which allow for an *ab initio* ligand field theory analysis (Atanasov *et al.*, 2012 ▸). As a result, we obtain an unrivaled understanding of the molecular magnetic properties of **1** and **2**.

## 2. Experimental methods   

### 2.1. Synthesis   

Crystals of **1** and **2** were obtained from synthesis and recrystallization according to the published procedure (Vaidya *et al.*, 2018 ▸).

### 2.2. Synchrotron X-ray diffraction and multipole modeling.   

**Figure 1:**
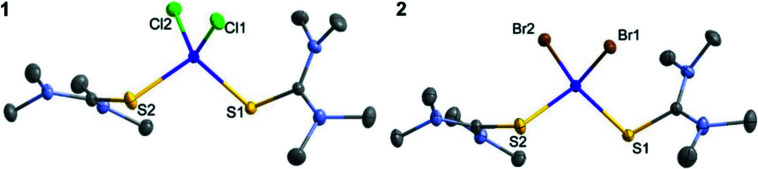
*ORTEP* drawings of **1** (left) and **2** (right). Thermal ellipsoids showing 90% probability surfaces. Hydrogen atoms have been omitted for clarity.

**Figure 2:**
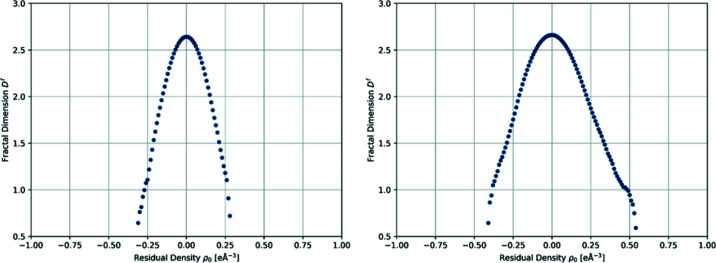
Fractal dimensionality plots for **1** (left) and **2** (right).

Single crystals of **1** and **2** of suitable size for the synchrotron beam were mounted on a Huber goniometer head via a glass-fiber and using glue. The crystal quality was tested at room temperature and subsequently cooled to low temperature. Data were collected at beamline BL02B1 at SPring-8, which is equipped with a Pilatus3 X CdTe 1M detector and a gaseous He-cooling device. The datasets consist of 180° omega-scans in steps of 0.5° with χ fixed at 0, 20 and 45° and with 2θ = 0 and 20° (scan speeds of 2 and 4° s^−1^ for **1**, and 1 and 2° s^−1^ for **2**), thus six runs for each crystal. The detector frames are converted to Bruker format, followed by a Lorentz and polarization correction, and the raw images are then integrated using *SAINT+* (v8.38A). Subsequently, integrated intensities are corrected for absorption and other effects in *SADABS* (Krause *et al.*, 2015 ▸), and resulting unmerged data without application of an error-model are then finally merged in *SORTAV* (Blessing, 1997 ▸). Final *hkl* files are used to solve and refine structures in *SHELXT* (Sheldrick, 2015*a* ▸) and *SHELXL* (Sheldrick, 2015*b* ▸), respectively. The resulting structures are imported into *XD* (Volkov *et al.*, 2006 ▸) and a multipole model is incrementally built, until we reached the final model, which for **2** included anharmonic parameters for bromium (Herbst-Irmer *et al.*, 2013 ▸). We emphasize here the importance of using radial functions based on Hartree–Fock calculations for the metal center, in order to obtain physically reliable *d*-orbital populations (Damgaard-Møller *et al.*, 2020*c* ▸). Further details about the multipole model are deposited in the supporting information; Table 1 ▸ contains essential crystallographic results. Fig. 1 ▸ shows the molecular structures, while Fig. 2 ▸ shows the fractal dimensionality plots (Meindl & Henn, 2008 ▸) for both compounds, indicating the high reliability of the final models. For the ensuing discussion of the *d*-orbital populations, it is important to clarify the choice of local coordinate system. In both complexes, we have defined the *z* axis to bis­ect one *X*—Co—S angle such that it points from Co towards the tmtu ligands. There are several reasons for this choice: (1) the deformation density maps around Co show clear maxima corresponding to these directions; (2) it corresponds to directions obtained from theoretical calculations; (3) the perception of the coordination sphere of Co as a distorted tetrahedron fits best with this direction as the unique axis in approximate *D*_2*d*_ point symmetry.

### 2.3. Polarized neutron diffraction   

Flipping ratio measurements based on experiments with single-crystal PND, and its intimate connection with the magnetization density were first explained in 1959 by Nathans *et al.* (1959 ▸). The period from 1970 to 1990 saw many experimental studies with particular emphasis on spin-only 3*d* metal molecular complexes to derive spin density distributions, with substantial contributions from Figgis and Reynolds (Daul *et al.*, 1988 ▸; Figgis *et al.*, 1988 ▸, 1987 ▸, 1983 ▸, 1982 ▸) and others (Forsyth, 1977 ▸). Focus was, at that time, on complexes with little or no orbital angular momentum, for which the induced magnetization density vector aligns perfectly with the external field. The effect of significant orbital angular momentum is, however, that we can no longer assume such collinearity. The direct link between flipping ratios and magnetization density thus becomes invalid and the latter cannot be experimentally recovered. Under such circumstances, Brown and Gukasov showed that flipping ratio measurements can instead be used to retrieve a local atomic susceptibility tensor (Gukasov & Brown, 2002 ▸) which models this absence of collinearity.

Recently, their approach has been extended to study the magnetic anisotropy of paramagnetic compounds in polycrystalline samples (Kibalin & Gukasov, 2019 ▸; Gukasov & Brown, 2010 ▸). Paramagnetic complexes are at the center of SMM research, and this development is therefore of enormous importance. Until recently, the lack of appropriate software made the powder PND technique practically inapplicable to data refinement, but a library of dedicated computer code is now available and, having established an initial proof-of-concept, the technique can be considered ready for application to novel systems (Kibalin & Gukasov, 2019 ▸). We envisage that this approach will receive a massive boost when the European Spallation Source becomes operational in a few years time.

A detailed description of the theoretical basis for the PND method for both powder and single crystals is provided in the supporting information.

PND measurements for this work for both the powder- and single-crystal studies were performed at the thermal polarized neutron lifting counter diffractometer 6T2 (LLB-Orphée, Saclay). Neutrons were monochromated to a wavelength of 1.4 Å by a vertically focusing graphite crystal and polarized by a supermirror bender. The polarization factor *P* of the beam was 0.95 for the powder measurements on **1** and 0.78 for the single-crystal measurements on **2**. Details of the powder data collection are provided by Kibalin & Gukasov (2019 ▸) and details on the data reduction and refinement are supplied in the supporting information. The powder diffraction patterns for **1** were measured at a magnetic field of 1 T and a temperature of 2 K using a position-sensitive detector. Single-crystal measurements for **2** were made in the magnetic field of 1 T at 3 K, and the flipping ratios were collected for five different sample orientations. Flipping ratios for compound **2** were then extracted from the raw images by employing the in-house data reduction suite at the LLB.

The refinement of susceptibility tensors both for single-crystal and powder PND data were performed with the newly developed software library *Cryspy* (v. 0.5.8) through the *Cryspy Editor* (v.1.5.6), both available for Python3.X through PyPI (Kibalin & Gukasov, 2019 ▸).

### 2.4. Theoretical calculations   

*Ab initio* calculations were performed using the *ORCA* software (4.1; Neese, 2012 ▸, 2018 ▸) with the solid-state geometry of complexes **1** and **2** obtained from synchrotron X-ray diffraction at 20 K. CASSCF(7,5) (Malmqvist & Roos, 1989 ▸) and subsequent NEVPT2 (Angeli *et al.*, 2001 ▸) correction was performed including all the ten quartet *S* = 3/2 states and using the Douglas–Kroll–Hess triple-ζ DKH-def2-TZVP basis set (Schäfer *et al.*, 1992 ▸, 1994 ▸; Weigend & Ahlrichs, 2005 ▸). Including the 40 doublet states (*S* = 1/2 states) did not change the results. The *AILFT* (Atanasov *et al.*, 2012 ▸, 2013 ▸) program was used to get an estimated *d*-orbital splitting of the compounds. The SOC was accounted for on the basis of non-relativistic configuration interaction eigenstates using quasi-degenerate perturbation theory. Relativistic electron densities used in the analysis were obtained from the ground Kramers doublet and first exited Kramers doublet. Projection of the two lowest Kramer doublets onto an *S* = 3/2 pseudo spin furthermore allowed for the extraction of the SH parameters *g_x_*, *g_y_*, *g_z_*, *D* and *E*.

## 3. Results and discussion   

We start with an analysis of the crystal structure. The Co*X*_2_S_2_ coordination geometries in **1** and **2** obtained from the crystal structures at 20 K are summarized in Table 2 ▸. The deviation from tetrahedral symmetry is quite complicated to quantify, and the typical shape index descriptor (Pinsky & Avnir, 1998 ▸) does not add valuable insight. There is little asymmetry (<0.03 Å) in the bond lengths from cobalt to both *X* and S, internally in both compounds. Obviously, the bond angles show a strong deviation from tetrahedral geometry. Closer inspection suggests that two of three angles involving *X*2—Co are significantly larger than 109.47° and the third is quite close to this value, whereas two of three angles involving *X*1—Co are much smaller. Thus, we may describe the distortion of the initial ideal tetrahedral coordination sphere as one where we view down the *X*2—Co axis and squeeze S1 and *X*1, while S2 is more or less left in place. However, we choose instead to view the coordination sphere as a compressed tetrahedron with approximate *D*_2*d*_ symmetry, where the unique axis is the one that bis­ects the *X*1—Co—S1 angle. This axis is the one we use as the *z* axis in the definition of the orbital functions in the multipole modeling (*vide infra*).

### 3.1. Theoretical results   

Before we describe the experimental results (Section 3.2 and onwards), we look at the calculated electronic structures of **1** and **2**, obtained using the *ORCA* program suite from CASSCF(7,5) and subsequent NEVPT2 correction to the energies. Two main results from the calculations will be described in turn in the following: (1) the *AILFT* orbitals provide an intuitive interpretation and prediction of the magnetic anisotropy; (2) the *g*, *D* and *E* values, which quantify the magnetic anisotropy, and can be compared with the experimental measurement of the magnetic susceptibility tensors. In addition, Section 3.3 contains a comparison of the theoretically calculated electron density (ED) and the experimentally derived ED.

**Figure 3:**
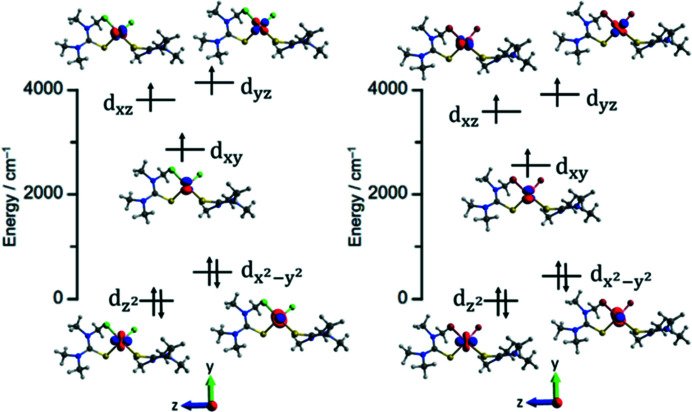
*AILFT* orbital energy diagrams for **1** (left) and **2** (right), The main *d*-orbital components and the corresponding isosurface plots are also provided.

(1) The AILFT analysis of an *ab initio* calculation provides orbitals independent of the input molecular coordinate system, thus providing the most consistent orbitals. As already discussed, both **1** and **2** are best described as distorted tetrahedral complexes (see above). Therefore, we expect to have the *e*-type (in *T_d_* symmetry) orbitals (*i.e.* 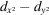 and 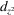) stabilized, and the *t*_2_-type orbitals (*i.e.* *d_xy_*, *d_xz_* and *d_yz_*) destabilized. The resulting *AILFT* orbitals and their energies are depicted in Fig. 3 ▸. Several indicators suggest that the chosen assignment is correct. First of all, it matches the results of several other distorted tetrahedral Co(II) complexes, in which the 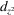 orbital is stabilized, and has its lobes pointing in the direction of the narrowest *L*—Co—*L* bis­ecting angle (Carl *et al.*, 2015 ▸; Rechkemmer *et al.*, 2016 ▸; Zadrozny & Long, 2011 ▸; Zadrozny *et al.*, 2013 ▸). Secondly, this choice stabilizes *d_xy_* compared with the other *t*_2_-orbitals, and the coupling of 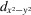 and *d_xy_* would predict easy-axis type anisotropy in the direction of the *z* axis, which is also true in the present case. The last indication that these are ‘good’ orbitals is provided by the composition of the CASSCF wavefunction, which in the given coordinate system consist of fairly pure single Slater-determinants (91.6% 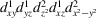 for the ground state and 84.0% 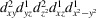 for the first excited state in **1** and 89.7% 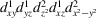 for the ground state and 76.2% 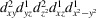 for the first excited state in **2**). We note that the orbital ordering reported for both complexes in the earlier report (Vaidya *et al.*, 2018 ▸) are slightly different from the orbital ordering reported here. This is because, in the earlier paper, we reported the CASSCF ground state wavefunction which is multideterminant in character, whereas in this manuscript we report *AILFT* orbital ordering. However, we noticed the same orbital ordering (as we notice in this manuscript) when we compute the *AILFT* orbitals.

(2) The pseudo-spin 3/2 SH approximation allows for the extraction of the magnetic anisotropy from the *ab initio* calculations. The eigenvectors for the *D*- and *g*-tensor are almost parallel, and the eigenvalues as well as the directions of the vectors with respect to the coordinate system chosen for the *AILFT* orbitals above are found in Table 3 ▸.

**Figure 4:**
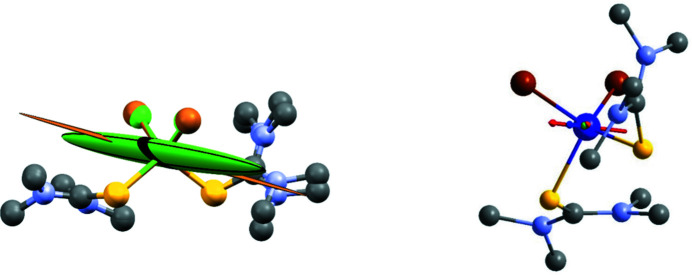
(*a*) Atomic susceptibility tensors for Co derived from PND for **1** and **2**, illustrated as thermal ellipsoids. The eigenvalues of the susceptibility tensors are −0.29 (1), 0.86 (1), 4.07 (1) μ_B_/*T* for **1** and 0.03, 0.11, 2.38 µ_B_/*T* for **2**. The negative eigenvalue is shown as positive and the ellipsoids have been scaled arbitrarily for the purpose of visualization. The ellipsoids for Co are colored green and brown in **1** and **2**, respectively. (*b*) Magnetic easy axes from theory and experiment on **1** and **2**. The easy axes directions are shown as red, green, blue and orange arrows for Cl-experimental, Br-experimental, Cl-theoretical and Br-theoretical, respectively. All arrows are overlaid on the molecular structure of **2** using the same procedure of RMSD-minimization as described in the main text. Atoms are colored purple (Co), yellow (S), gray (C), blue (N), brown (Br), green (Cl).

The *D*-tensor gives *D* = −18.57 cm^−1^, ∣*E*/*D*∣ = 0.067 for **1** and *D* = −17.75 cm^−1^, ∣*E*/*D*∣ = 0.086 for **2**. The easy axis of the compounds (*i.e.* characterized by *D_zz_* or *g_z_*) is not parallel with the chosen molecular *z* axis for the *AILFT* orbitals, but forms an angle with this *z* axis of 15° for **1** and 11° for **2** (see Fig. 4 ▸). In the case of pure orbitals, the easy axis aligns perfectly along the molecular *z* axis, and the observed easy axis thus shows the inadequacy of the *d*-orbital diagram to accurately predict the magnetic anisotropy. This is caused by the multi-configurational nature of the ground state and first-excited state, where only about 90% is a pure Slater determinant, and the remaining 10% of the ground state allows for change in the direction of the easy axis. Nevertheless, the *d*-orbital scheme gives a useful and intuitive explanation for both sign and magnitude of the magnetic anisotropy, providing valuable guidelines for a synthetic chemist in the search for new transition metal SMMs (Ruamps *et al.*, 2013 ▸; Gomez-Coca *et al.*, 2013 ▸).

Previous studies have found clear evidence that distortion of the tetrahedral geometry around Co(II) can lead to strong magnetic anisotropy (Rechkemmer *et al.*, 2016 ▸; Tripathi *et al.*, 2019 ▸; Vaidya *et al.*, 2018 ▸, 2017 ▸; 2016 ▸; Legendre *et al.*, 2021 ▸). This has been explained by the appearance of a strong splitting of the *d*-orbitals of *t*_2_-symmetry and, to a smaller extent, of the *e*-symmetry orbitals, with the great benefit that the energies of the 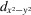 and *d_xy_* orbitals approach each other. Perhaps surprisingly, it is often observed that the 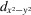 is destabilized relative to 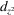 upon tetrahedral compression. However, regardless of the order of these two latter orbitals, a large and negative *D*-value is predicted and most often realized. As explained above, the geometrical distortions of the coordination spheres in **1** and **2** are rather complicated and the unique axis of magnetization is extremely difficult to rationalize from structural considerations alone. Similarly, the theoretical result indicates a significant deviation of the anisotropy axis away from the molecular *z* axis. We have therefore determined the direction and the associated anisotropy of the unique axis of magnetization using PND for both compounds, as described in Section 3.2.

### 3.2. Experimental results: PND   

The experimental susceptibility tensors, given in the normalized reciprocal coordinate system, 
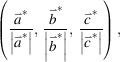
have the form
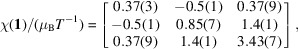


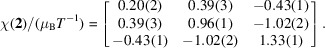
Fig. 4 ▸ shows these susceptibility tensors as ellipsoids overlaid on the molecular structures of **1** and **2** and with the structures rotated so as to minimize the root-mean-square distance (RMSD) between the first coordination sphere and Co atoms in the two structures. The final RMSD value obtained from this procedure is 0.0942 Å. It is evident that the experimental easy axis of magnetization in both compounds approximately bis­ects the X1—Co—S1 angles, with deviations from the Cl1—Co—S1 and Br1—Co—S1 bis­ectors being 24.6 and 10.8° for **1** and **2**, respectively, and an approximate deviation between the two experimental easy axes of 23.3° [Fig. 4 ▸(*b*)]. For comparison, the angle between the two theoretical easy axes of **1** and **2** is calculated to be 12.4°. The extent of axiality, which we define here as the ratio of the largest eigenvalue to the average of the two smallest eigenvalues, is 14.8 for **1** and a staggering 33.1 for **2**. An even better metric for the axiality, using the susceptibility tensors for these two structures might however be what we here call an ‘effective’ ∣*E*/*D*∣, based on the relationship 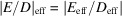, where 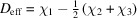, 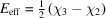; and χ_1_, χ_2_ and χ_3_ are the largest, intermediate and smallest eigenvalues of the tensor. This metric gives 0.15 and 0.018 for **1** and **2**, respectively. The theoretically calculated value of ∣*E*/*D*∣ for **1** is roughly half of what we find here experimentally. The experimental extent of axiality is quite extreme for **2**, and thus the effective ∣*E*/*D*∣ is much smaller for this compound than what is found theoretically.

In addition to a comparison based on their eigenvalues, the susceptibility tensors also allow us to compare the easy axis directions obtained from PND with the calculated ones [Fig. 4 ▸(*b*)]. We see a striking agreement between the calculated and measured directions, particularly for **2**, with deviations between the theoretical and experimental result of only 12.1 and 1.8° for **1** and **2**, respectively.

### 3.3. Experimental results: electron density   

On the basis of this unique result from the PND analysis, we now discuss the experimental and theoretical ED analysis. As mentioned above, the *z* axis of the local coordinate system that defines the multipolar functions is chosen to match the direction of the *AILFT* orbitals from *ab initio* theory (*i.e.* along the angle bis­ector of *X*1—Co—S1). The *y* axes point between the two halogens, and the *x* axes approximately bis­ect the *X*1—Co—S2 angle. With this convention, we obtained the experimental *d*-orbital populations (Table 4 ▸) from the multipole parameters using the transformation matrix given by Holladay *et al.* (1983 ▸). To discuss the values obtained, it is important to know what they represent. When looking at the simplest description of the system, with pure *d*-orbitals and electrons placed according to the Aufbau principle (see Fig. 3 ▸), we expect the populations to be 1.00 in the *t*_2_ antibonding orbitals and 2.00 in the *e* non-bonding orbitals. This is obviously a too-simplistic description of the true ED around cobalt, as it is affected by metal–ligand electron transfer and SOC, and perhaps also geometrical distortions and relaxation effects. Next, we discuss the potential impact on the *d*-orbital populations of the two former effects.

Metal–ligand electron transfer/covalent bonding is a well known effect for transition metal compounds. Its influence on *d*-orbital populations is due to the fact that ligands of the same symmetries form bonding and antibonding MOs with contributions from some of the *d*-orbitals (Figgis & Hitchman, 2000 ▸). In approximate tetrahedral symmetry as in **1** and **2**, the *t*_2_-orbitals [*d*(*xy*), *d*(*yz*), *d*(*xz*)] have the same symmetry as the symmetry-adapted linear combination of the four σ-donating lone pairs on the ligands. The bonding orbitals of these are fully occupied and primarily constituted by ligand orbitals, and not part of the conventional active space. However, due to the finite ligand contribution to these MOs, they will add modest ED near the metal center, and therefore it is to be expected that the average *d*-orbital population of the *t*_2_-orbitals is larger than 1.000. Furthermore, this effect would add more electrons to the *d_yz_* and *d_xz_* orbitals than to *d_xy_* since the latter is slightly stabilized relative to the two former (see Fig. 3 ▸) and thus experience less overlap with the ligand orbitals. This is clearly expressed in the experimental *d*-orbital populations of **2**, which show a significantly larger population of *d_yz_* and *d_xz_* compared with the *d_xy_* orbital, and thus indicates that **2** has a much larger degree of metal–ligand electron transfer compared with **1**. This is backed by a topological analysis of the ED (Table 5 ▸) which shows that the total energy density – to some extent a measure of covalency in a chemical bond (Gatti, 2005 ▸) – is consequently less positive for **2** than for **1**, indicating that the σ-donation from the halide is indeed higher for the Br-complex. However, in the populations for the other models shown in Table 4 ▸, the *d_xy_* orbital is populated more than the *d_yz_* and *d_xz_* orbitals. We may ascribe this to the effects of SOC, as explained below.

SOC is an effect that couples ground and excited states, and thus in effect leads to withdrawal of electrons from the doubly occupied orbitals, and insertion into some of the singly occupied orbitals. In this specific case, the ground-state Kramers doublet consists of 97% of the spin-orbit free ground state (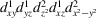) and 3% of the first-excited spin-orbit free state (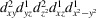) in both **1** and **2**. Given the spin-orbit free states mentioned earlier, the resulting *d*-orbital populations of *d_xy_* and 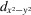 would be 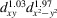, thus in addition to the electrons from metal–ligand interaction, the population of *d_xy_* would be increased due to SOC.

There is thus a competition between the metal–ligand covalency on the one hand, which increases the populations of *d_xz_*, *d_yz_* more than it does *d_xy_*, and SOC on the other hand, which effectively moves electrons from 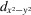 to *d_xy_*. We have recently shown how the effect of the SOC can be used to quantify the ZFS from experimental *d*-orbital populations (Damgaard-Møller *et al.*, 2020*c* ▸). However, in the present case with much smaller SOC, the quantification is not reliable. We also note that the population of 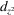 is less than 2.0, which is very likely due to 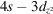 mixing, which is possible as they have the same symmetry.

To evaluate the expected size of the two opposing effects described above, we obtained *d*-orbital populations from theory (Table 4 ▸), by refinement of theoretical structure factors calculated from the relativistic, SOC ground state of compounds that provide the best possible description of real-world ED (Genoni, 2020 ▸; Gao *et al.*, 2020 ▸). The relativistic SOC electron densities extracted from the *ab initio* calculation (*i.e.* the ED of the lowest two Kramer doublets) were combined into one ED using a Boltzmann averaging at 20 K, and this was modeled using the Hansen–Coppens multipole formalism (Hansen & Coppens, 1978 ▸). Table 4 ▸ shows that there is a clear, albeit small, difference suggesting that the σ-donation is larger in **2** than in **1**, while the identical populations of 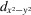 in **1** and **2** suggest that the effect of SOC is identical, not surprising given the nearly identical energy level differences seen in Fig. 3 ▸.

## 4. Conclusions   

We have quantified the magnetic anisotropy in the form of atomic susceptibility tensors for two isostructural four-coordinate Co(II) complexes using PND. The results clearly show highly axial and comparable magnetic anisotropy for both compounds, with unique axes aligned approximately along the direction of the molecular *z* axis, from Co and bis­ecting the *X*1—Co—S1 angle. Exceptionally, this study shows for the first time that PND studies using powder samples can provide atomic susceptibility tensors of comparable quality to more traditional single-crystal studies. Using the *z* axis direction, an *AILFT* analysis of the CASSCF results offer an explanation for the magnetic anisotropy showing the increased proximity of *d_xy_* and 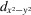 orbitals relative to an ideal tetrahedral coordination. The small and opposite effects of metal–ligand covalency and spin-orbit coupling are identified in the electron density models refined using very high-resolution, low-temperature synchrotron X-ray diffraction data. Combined, the range of experimental results unanimously show that the σ-donation to the Co(II) ion is larger in **2** with Br-ligands than in the Cl-ligated compound **1**.

## 5. Related literature   

The following references are cited in the supporting information: Clark *et al.*(2007 ▸); Gukasov *et al.* (2007 ▸); Politzer & Murray (2019 ▸).

## Supplementary Material

Crystal structure: contains datablock(s) cocl, cobr. DOI: 10.1107/S2052252521008290/lt5038sup1.cif

Additional information, figures and tables. DOI: 10.1107/S2052252521008290/lt5038sup2.pdf

CCDC references: 2104887, 2104888

## Figures and Tables

**Table 1:** Crystallographic details for the synchrotron data collections and refinements of **1** and **2**

	**1**	**2**
Empirical formula	C_10_H_24_Cl_2_CoN_4_S_2_	C_10_H_24_Br_2_CoN_4_S_2_
Formula weight (g mol^−1^)	394.28	483.18
Crystal size (mm)	∼0.1 × 0.1 × 0.1	∼0.1 × 0.1 × 0.1
Crystal system	Monoclinic	Monoclinic
Space group	*P*2_1_/*n*	*P*2_1_/*n*
λ (Å)	0.2486	0.2486
*a* (Å)	9.9071 (4)	9.798 (4)
*b* (Å)	12.7019 (5)	12.961 (8)
*c* (Å)	14.1556 (6)	14.636 (6)
β (°)	92.824 (2)	92.363 (2)
*V* (Å^3^)	1779.16 (13)	1856.9 (2)
*Z*	4	4
*F*(000)	820	964
*T* (K)	20	20
ρ (g cm^−3^)	1.472	1.732
μ (mm^−1^)	0.094	0.275
*T*_max_, *T*_min_	0.9576, 0.9030	0.7444, 0.6698
*N*_meas_, *N*_uniq_	649992, 68940	736910, 38434
Completeness	100	100
*R*_int_	0.074	0.083
Resolution (included data) (Å)	0.4	0.4
*R*_*w*_(*F*^2^), [*I* > 2σ(*I*)]	0.031	0.014
*R*(*F*), *R*(*F*^2^), all data	0.07, 0.03	0.056, 0.029
Goodness of fit	1.021	0.993

**Table 2:** Selected geometrical parameters extracted from the 20 K crystal structures of **1** and **2**

	**1**	**2**
*d*(Co—*X*1) (Å)	2.26935 (5)	2.41603 (6)
*d*(Co—*X*2) (Å)	2.25542 (5)	2.40284 (6)
Δ(Co—*X*) (Å)	0.01393	0.01319
*d*(Co—S1) (Å)	2.33625 (5)	2.32758 (7)
*d*(Co—S2) (Å)	2.36393 (5)	2.34945 (8)
Δ(Co—S) (Å)	0.02768	0.02187
∠S1—Co—S2 (°)	106.304 (2)	106.744 (3)
∠*X*1—Co—*X*2 (°)	114.186 (2)	113.804 (2)
∠*X*1—Co—S1 (°)	106.089 (2)	103.895 (2)
∠*X*1—Co—S2 (°)	105.728 (2)	105.132 (2)
∠*X*2—Co—S1 (°)	114.208 (2)	117.482 (2)
∠*X*2—Co—S2 (°)	109.700 (2)	108.884 (2)

**Table 3:** Eigenvalues of the calculated *D*- and *g*-tensors for **1** and **2** Elements of *D* are given in cm^−1^ and *g* is unitless. Note that we use here the criterion ∣*D*∣ ≥ 3*E* ≥ 0 in contrast to the definition 1/3 ≥ *E*/*D* ≥ 0 used in *ORCA*.

	Eigenvalues (traceless)	*X*	*Y*	*Z*
**1**				
*D_xx_*	7.43	0.71	−0.67	−0.23
*D_yy_*	4.94	−0.66	−0.74	0.09
*D_zz_*	−12.38	0.23	−0.09	0.97
*g_x_*	2.25	0.75	−0.62	−0.25
*g_y_*	2.28	−0.62	−0.78	0.08
*g_z_*	2.48	0.24	−0.09	0.97
				
**2**				
*D_xx_*	7.44	−0.82	−0.57	−0.10
*D_yy_*	4.40	−0.57	0.80	0.16
*D_zz_*	−11.83	0.01	−0.19	0.98
*g_x_*	2.26	−0.84	−0.53	−0.09
*g_y_*	2.30	−0.53	0.82	0.18
*g_z_*	2.49	0.02	−0.20	0.98

**Table 4:** *d*-orbital populations obtained from experimental multipole parameters for **1** and **2**. The values in parentheses are the percentage populations of the entire 3*d* shell

Symmetry	*d*-orbital	**1** (exp)	**1** (theory)	**2** (exp)	**2** (theory)
*t*_2_	*d*(*yz*)	1.12 (16.0)	1.06 (15.0)	1.26 (16.9)	1.08 (15.2)
	*d*(*xz*)	1.08 (15.4)	1.07 (15.1)	1.27 (17.1)	1.10 (15.5)
	*d*(*xy*)	1.11 (15.9)	1.07 (15.1)	1.13 (15.2)	1.06 (15.0)
*e*	*d*(*x*^2^ − *y* ^2^)	1.86 (26.6)	1.95 (27.7)	1.98 (26.6)	1.95 (27.4)
	*d*(*z*^2^)	1.82 (26.1)	1.91 (27.1)	1.79 (24.1)	1.91 (26.9)
	SUM	6.99	7.06	7.43	7.10

**Table 5:** Topological analysis of the electron density First line is from **1**, second line from **2**. Units are eÅ^−3^ for ρ_bcp_, eÅ^−5^ for ∇^2^ρ_bcp_, Å for distances (*d*); and energy densities *G*, *V* and *H* are given in hartree au^−3^.

Bond	Model	ρ_bcp_	∇^2^ρ_bcp_	*d*_1-2_	*d*_1-bcp_	*d*_2-bcp_	*G*	*V*	*H*
Co—*X*1 (long)	Cl-exp	0.34	7.03	2.270	1.029	1.241	0.460	−0.428	0.032
	Cl-theo	0.36	7.51	2.269	1.018	1.251	0.499	−0.472	0.027
	Br-exp	0.42	3.59	2.417	1.108	1.310	0.351	−0.328	0.023
	Br-theo	0.32	5.76	2.416	1.049	1.366	0.386	−0.369	0.017
Co—S1 (short)	Cl-exp	0.33	5.86	2.337	1.041	1.295	0.398	−0.385	0.013
	Cl-theo	0.35	6.28	2.336	1.033	1.303	0.435	−0.430	0.005
	Br-exp	0.50	4.16	2.327	1.074	1.252	0.405	−0.413	−0.008
	Br-theo	0.37	6.36	2.329	1.032	1.298	0.452	−0.459	−0.007
Co—*X*2 (short)	Cl-exp	0.36	7.41	2.256	1.021	1.235	0.494	−0.469	0.025
	Cl-theo	0.38	7.81	2.256	1.012	1.243	0.523	−0.500	0.023
	Br-exp	0.41	3.68	2.406	1.111	1.295	0.383	−0.377	0.006
	Br-theo	0.32	5.78	2.404	1.048	1.356	0.390	−0.374	0.015
Co—S2 (long)	Cl-exp	0.30	5.37	2.364	1.054	1.311	0.356	−0.336	0.020
	Cl-theo	0.33	5.87	2.364	1.044	1.320	0.399	−0.387	0.012
	Br-exp	0.44	3.76	2.348	1.098	1.250	0.386	−0.374	0.011
	Br-theo	0.36	6.25	2.349	1.035	1.315	0.437	−0.437	0.000
