# Supplementary material for: Quantifying magnetic anisotropy using X-ray and neutron diffraction
Source: IUCrJ. 2021 Sep 1;8(Pt 5):833–41. doi: 10.1107/S2052252521008290 (PMC8420765; doi:10.1107/S2052252521008290)
Supplement: Supplementary file 2 [file m-08-00833-sup2.pdf]

IUCrJ

Volume 8 (2021)

Supporting information for article:

Quantifying magnetic anisotropy using X-ray and neutron diffraction

Emil Andreasen Klahn, Emil Damgaard-Møller, Lennard Krause, Iurii Kibalin, Arsen Gukasov, Shalini Tripathi, Abinash Swain, Maheswaran Shanmugam and Jacob Overgaard

Supporting Information for

Quantifying Magnetic Anisotropy Using X-ray and Neutron diffraction

*Emil Andreasen Klahn, Emil Damgaard-Møller, Lennard Krause, Iurii Kibalin, Arsen Gukasov, Shalini Tripathi, Abinash Swain, Maheswaran Shanmugam and Jacob Overgaard**

a) Department of Chemistry, Aarhus University, Langelandsgade 140, Aarhus C, 8000, Denmark
b) LLB, CEA, CE de Saclay, Gif sur Yvette, 91191, France
c) Department of Chemistry, IIT Bombay, Powai, Mumbai, Maharashtra, 400076, India

Correspondence email: jacobo@chem.au.dk

Contents

Contents 1

Details about multipole modeling using synchrotron diffraction data 3

Coordination to the X-atoms. 4

Comparison of different orbital decomposition schemes 6

Nuclear structure determination of $CoBr_2(tmtu)_2$ 8

Data description and refinement of powder polarized neutron diffraction data $CoCl_2(tmtu)_2$..... 9

Data description and refinement of polarized neutron diffraction on $CoBr_2(tmtu)_2$........ 16

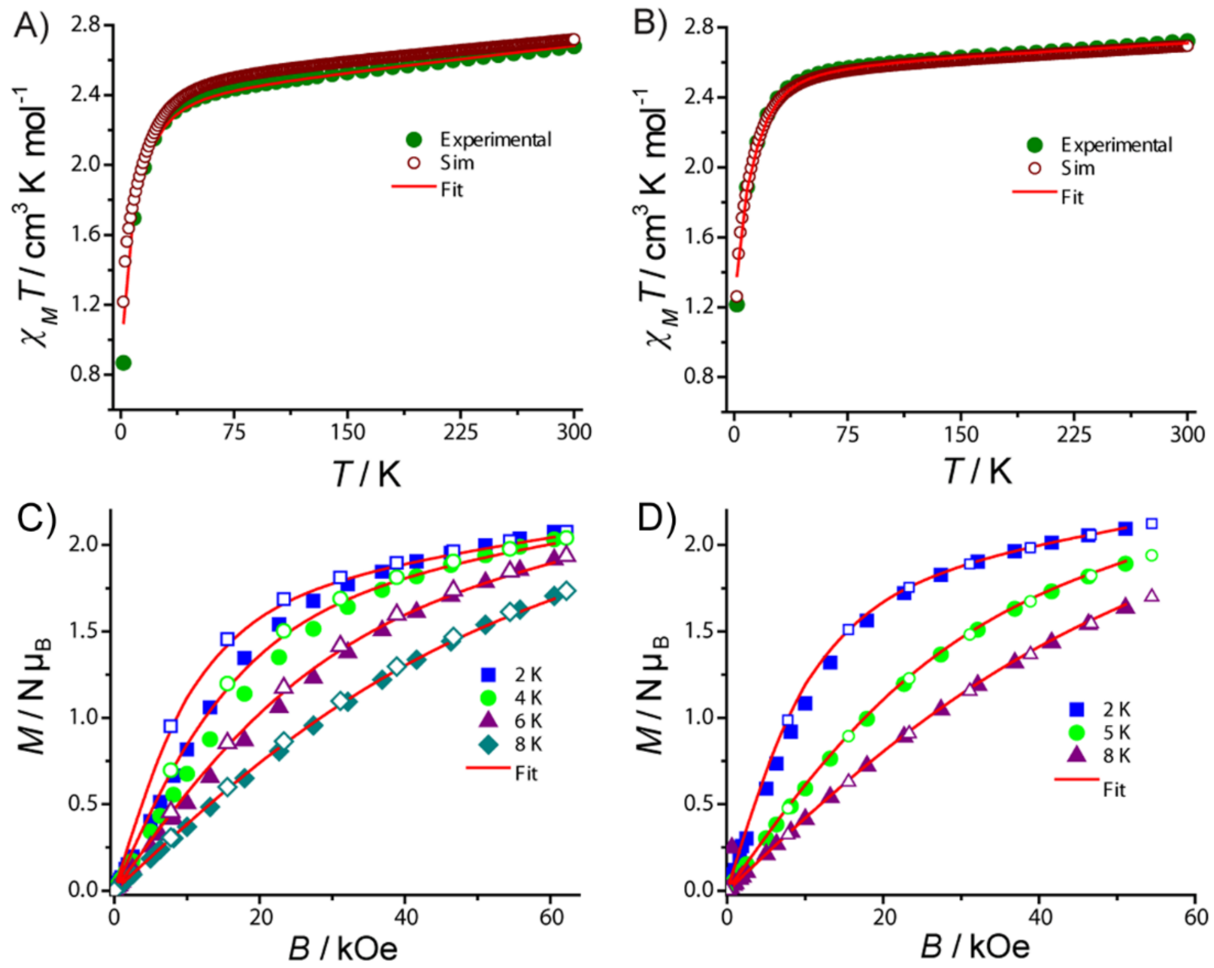

Figure S1. Variable temperature magnetic susceptibility measurement performed on polycrystalline sample of **1** and **2** (Panels A and B, respectively) in the presence of 1 kOe an external magnetic field and the field dependent magnetization measurements were performed at the indicated temperature (panels C and D, respectively). The solid red trace in all the plot denotes the best fit obtained using the SH parameters described in the main text. The wine-red open symbols in Panels A and B and the open symbols in Panels C and D is the simulation of the experimental magnetic data using the SH parameters extracted from *ab initio* calculations.

Details about multipole modeling using synchrotron diffraction data

For **1**, the data were integrated out to a resolution of 1.67 $Å^{-1}$. The data were truncated during the multipole modeling to a resolution of $\sin(\theta)/\lambda$ = 1.25 $Å^{-1}$, reducing the number of unique reflections to 29112, and a I(hkl)>2σ(I(hkl)) cutoff was applied reducing the number of used reflections to 25757.

For **2**, the data were integrated out to a resolution of 1.354 $Å^{-1}$. The data were truncated during refinement to a resolution of $\sin(\theta)/\lambda$ = 1.25 $Å^{-1}$, reducing the number of unique reflections to 30311, and a I(hkl)>2σ(I(hkl)) cutoff was applied reducing the number of used reflections to 26535.

Neutral scattering factors were used for all atoms. For Co, we found that the omission of the 4s electrons resulted in the best model. Thus, the 4s electrons were omitted from core and valence, and all valence electrons for Co were of 3d type. In **2**, the inclusion of third and fourth order Gram-Charlier coefficients was found to improve the fit. The resulting parameters were relatively small and the final parameters lead to negligible negative probability density. The maximum deviation from Hirshfeld rigid bond test was 6×10^{-4} $Å^2$ and 8×10^{-4} $Å^2$ for **1** and **2**, respectively, showing a very good deconvolution of thermal and electronic effects.

Coordination to the X-atoms.

For **1**, there are intramolecular C-H…Cl contacts. The important structural feature is the Co-X…H angle, which indicates if the H-atom is interacting with the σ-hole or the π-torus. In order of increasing distance, the values are for Cl1: 2.772, 2.936, 2.948, and 2.950 Å, and the associated Co-Cl…H angles are 78.3, 112.3, 166.9, 86.5°. For Cl2, there are only two less than 3 Å and they are 2.886 and 3.000 Å, with Co-Cl…H angles of 69.2 and 65.0°. For **2**, the shortest contact for Br1 is 2.942 Å followed by one of 3.000 Å. Two more are shorter than 3.2 Å. For Br2, the situation is similar, although the shortest contact is 2.832 Å, followed by 2.962 and 3.183 Å. For Br1, the angles are 70.8, 85.2, 138.4, and 134.5°, while for Br2 they are 75.0, 166.5, 138.1°, respectively. Thus, the shortest connections are always very acute and suggests an interaction with the π-torus, but the presence of rather short interactions at obtuse angles may indicate that there is no electrophilic σ-hole for Co-coordinated chlorine, in contrast to σ-bonded chlorine (Politzer & Murray, 2019, Clark *et al.*, 2007).

Table S1. AILFT orbital energies.

d-orbital	**1**	**2**
dz2	0	0
dx2y2	370.1	279.0
dxy	2681.6	2459.6
dyz	3513.6	3429.1
dxz	3832.7	3737.3

Comparison of different orbital decomposition schemes

In the first publication describing these complexes,(Vaidya *et al.*, 2018) a significantly different d-orbital diagram was reported (see Figure S2). This shows how important the orientation of the molecular coordinate system is, when it comes to the intuitive interpretation of the orbitals. The orbitals in Figure S2a represents a unitary transformation of the orbitals shown in Figure 3 in the main text, and thus describe exactly the same physical properties such as the ED, magnetic anisotropy, etc. However, the different choice of orbitals lead to a different CASSCF wave function. While we in this work have an almost pure single Slater determinant ground state, the orbitals in Figure S2a leads to a significantly more mixed ground state as seen in Figure S2b.

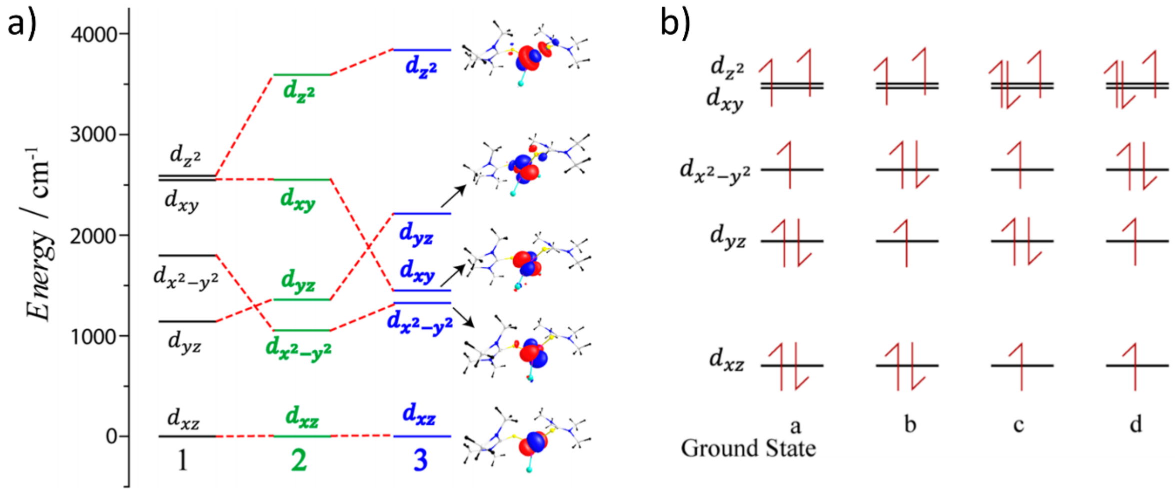

Figure S2. a) d-orbital diagram for **1**, **2** and **3** (where **3** is X = I, which is not included in our study). The visualized d-orbitals on the right is only shown for **1**. b) The ground state electron configuration for **1**, where a = 50%, b = 14%, c = 12% and d = 10%.

The previous report of d-orbitals and CASSCF wave function is thus equivalent to what we have reported in this study. However, we find the use of AILFT orbitals to give a more intuitive and consistent interpretation of the electronic structure. To showcase the equivalence between the previous study and our study, we have performed a separate ED refinement of **1**, where we have oriented the local coordinate system on cobalt to represent the d-orbitals reported in Figure S2a. Furthermore, we have calculated the expected d-orbital populations from the CASSCF ground state seen in Figure S2b, however, since the reported ground state only consist of 86% of the total ground state (due to exclusion of Slater determinants with a contribution of less than 5%) it is not possible to directly compare the numerical d-orbital populations. Instead, we compare the percentage populations, which correspond to distributing the remaining unreported electrons equally on all orbitals. If the results reported in the previous study represent the same ED as the results reported in this study, the calculated and experimental d-orbital populations

should be equal with this new coordinate system, and indeed, when looking at Table S2 the two d-orbital populations are very similar.

Table S2. Comparison of the theoretical populations of the d-orbitals from the previous study and experimental populations using the same coordinate system as reported in the previous study.

	Theoretical populations (Figure S2b)	Experimental populations (using coordinate system from Figure S2a)
$d(z^2)$	14.3%	15.2%
$d(xy)$	17.9%	18.4%
$d(x^2\text{-}y^2)$	18.3%	20.0%
$d(yz)$	24.6%	23.1%
$d(xz)$	24.9%	23.3%

Nuclear structure determination of $CoBr_2(tmtu)_2$

The nuclear structure of **2** was determined based on unpolarized neutron diffraction data collected on the KOALA diffractometer of the Australian Centre for Neutron Scattering (ACNS). The data were collected at a temperature of 10K using the Laue technique, with a maximum value of $\frac{\sin\theta}{\lambda}$ of $0.84\,Å^{-1}$. The structural model was refined by using a previously published X-ray diffraction model as starting point (Vaidya *et al.*, 2018) by loosening constraints on the hydrogen atoms such that both positions and anisotropic thermal parameters were refined freely for all atoms.

Table S3. Crystallographic details from refinement using unpolarized neutron data for **2**.

	**2**
Empirical Formula	$C_{10}H_{24}Br_2CoN_4S_2$
Formula Weight, g mol^{-1}	483.2
Crystal System	Monoclinic
Space Group	$P2_1/n$
λ, Å	Laue
T, K	10
a, Å	9.8432(19)
b, Å	12.942(3)
c, Å	14.629(3)
β, °	92.540(3)
V, Å^3	1861.8(7)
Z	4
F(000)	964
ρ, g cm^{-3}	1.724
N_{meas}	6711
Completeness, %	64.6
R1, %	7.36
wR2, %	12.21
Goodness of fit	1.164

Methodological background, data description and refinement of powder polarized neutron diffraction data $CoCl_2(tmtu)_2$

The powder PND (pPND) study of the susceptibility tensor is based on the measurement of the diffracted intensity that is distributed around the ring commonly known as the Debye rings or Debye cones. In this case the scattered intensities y_+ and y_- are represented as a function of a two independent variables: Bragg angle 2θ and polar angle φ of the cone base, measured for the two different polarization states of the incident neutron beam:

$$y_\pm(2\theta,\varphi) = S\sum_h m_h \cdot L \cdot P_h \cdot \langle I_\pm \rangle \cdot \psi_h(2\theta - 2\theta_h, \varphi) + b(2\theta,\varphi), \qquad (1)$$

where S is a scale factor, m_h is the multiplicity of reflection, L is the Lorentz factor, P_h is the density of (hkl) poles at the scattering vector (preferred orientation), $\psi_h(2\theta - 2\theta_h, \varphi)$ is the peak profile function normalized to unit area, and $b(2\theta,\varphi)$ is the background. The summation is done over all Bragg reflections h. Angular brackets indicate the averaging over crystallites orientations.

The powder averaging of $F^2_{M\perp}$, $(\boldsymbol{P} \cdot \boldsymbol{F}_{M\perp})$ terms in the integrated intensities (eq. 1) can be written as (Kibalin & Gukasov, 2019)

$$\langle F^2_{M\perp} \rangle = \frac{1}{2} B^2 [(\Sigma^2_{11} + 2\Sigma^2_{12} + \Sigma^2_{22}) \sin^2\alpha + 2(\Sigma^2_{13} + \Sigma^2_{23}) \cos^2\alpha]$$
$$\langle(\mathbf{P} \cdot \mathbf{F}_{\mathrm{M}\perp})\rangle = \mathrm{PB}\left(\frac{\Sigma_{11} + \Sigma_{22}}{2}\right) \sin^2\alpha \qquad (2)$$

Where α is the angle between the applied magnetic field and the scattering vector, which has the relationship $\cos^2\alpha = \cos^2\theta \sin^2\varphi$ to the Bragg angle θ and the cone base angle φ. The structure factor tensor Σ is $\mathrm{T}\chi(\mathbf{Q})\mathrm{T}^{-1}$, where T is the transformation matrix into a Cartesian coordinate system with the z axis parallel to the scattering vector $\mathbf{Q}$, and $\chi(\mathbf{Q})$ is the structure factor tensor in normalized reciprocal lattice in which the susceptibility parameters of magnetic atoms χ_a are defined. These equations describe the φ-dependent scattering along the Debye cones and are used in the 2D Rietveld refinement of the pPND-data. We note that the expression can also be applied generally, including for 1D data collected in a standard powder diffraction setup, in which case the scattering vector is in the scattering plane and $\varphi = 0$ ($\alpha = 90°$).

The powder diffraction data on **1** was measured by placing the powdered sample in an aluminium container and cooling the sample to 2 K. The full diffraction pattern was collected under a field strength of 1 T with a wavelength of 1.4 Å on the 6T2 diffractometer operated by the Laboratorie Léon Brillouin (LLB) on the Orphée reactor.(Gukasov *et al.*, 2007) The instrument has a 2-dimensional position sensitive detector with an acceptance area of 0.19 steradians (25˚ by 25˚). The diffraction pattern after background subtraction is shown in Figure S3. It was measured by

moving the detector from 17˚ to 65˚ in the horizontal plane in steps of 0.2˚ and measuring detector images with a 10 second exposure time for both parallel and antiparallel beam polarization. The images were combined to show the full data set in Figure S3, where the data has been shown as the sum of diffracted data measured with parallel and antiparallel beam polarization

$$I_+ + I_- \propto |F_N|^2 + \left|\boldsymbol{F}_{M,\perp}\right|^2$$

which is dominated by the nuclear scattering signal, F_N, and the difference of the diffracted data

$$I_+ - I_- \propto 4\boldsymbol{P} \cdot \mathcal{R}(F_N^* \boldsymbol{F}_{M,\perp})$$

which is non-zero, only when there is a magnetic signal and has the advantage that that the magnetic signal is enhanced by the presence of nuclear scattering. Both representations of the data show clear signs of preferred orientation because the diffraction signal in the individual Debye-Scherrer cones varies along the cone. We attribute this to the magnetic torque experienced by the magnetically anisotropic crystallites under the applied field. We note also that the strong signal around $\gamma = 35°$ in the sum pattern (Figure S3, top) is not due to the sample, but is due to the aluminium container, which has a strong Bragg-peak at this angle with a neutron wavelength of 1.4 Å. The non-magnetic nature of this aluminium peak is evident in the fact that the signal completely disappears in the difference pattern (Figure S3, bottom).

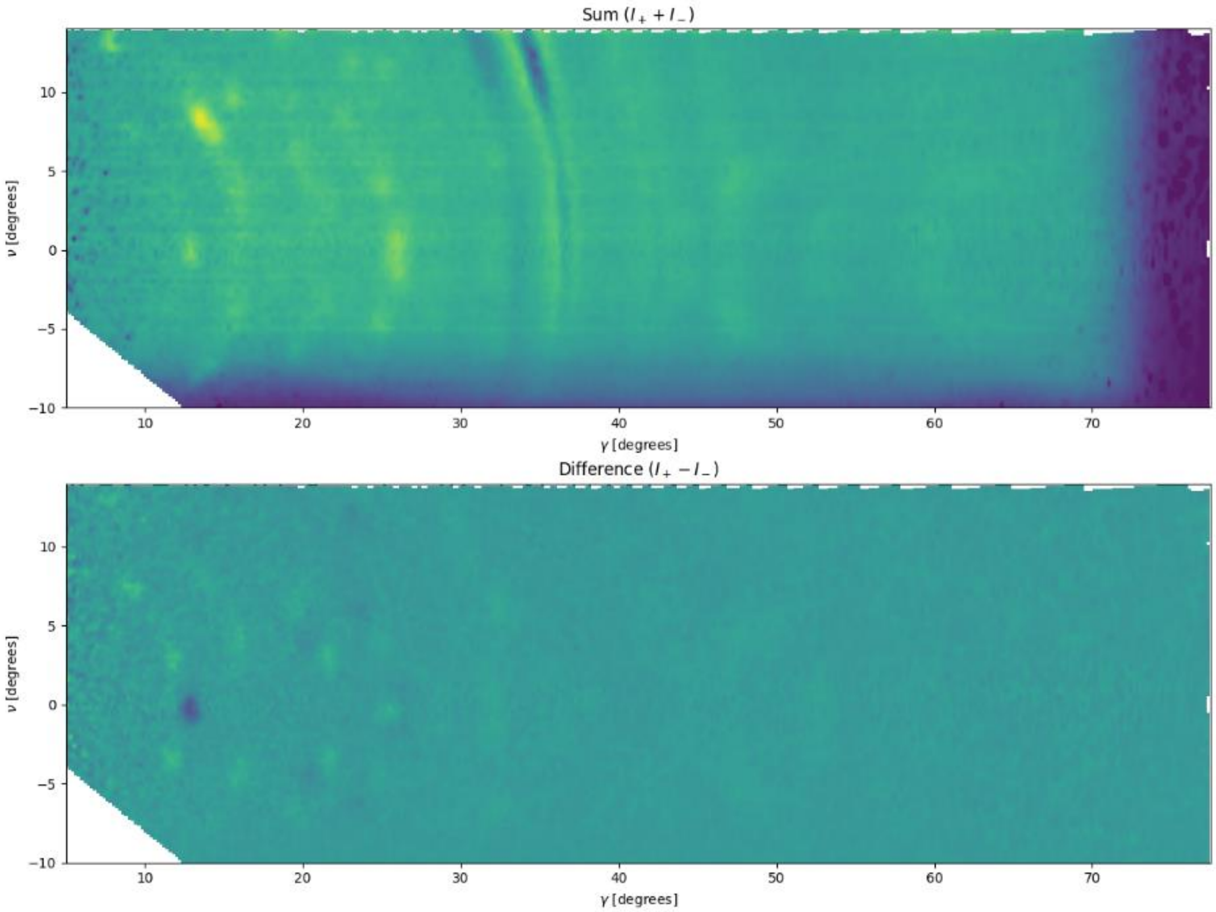

Figure S3: Powder diffraction pattern collected on the 6T2 diffractometer on **1**. Lighter color is more signal, darker color is less signal on an arbitrary scale. Top: Sum of diffraction patterns collected with both parallel and antiparallel beam polarizations. Bottom: Difference between diffraction patterns collected with parallel and antiparallel beam polarizations.

To refine the site susceptibility model against this data, we used the program package cryspy (v. 0.5.8) through the cryspy_editor (v. 1.5.6). The starting point for the refinement of the magnetic signal was the structure of **1** determined from high-resolution X-ray diffraction data and presented in the main text. The program uses the 2-dimensional data to fit against, but an intuitive way to summarize the result of the refinement is to collapse the pattern onto the 2θ-axis, as has been done in Figure S5. We note from comparison with a simulated powder diffraction pattern, shown in Figure S4, that the resolution in this experiment far from warrants a full structure refinement from the diffraction pattern.

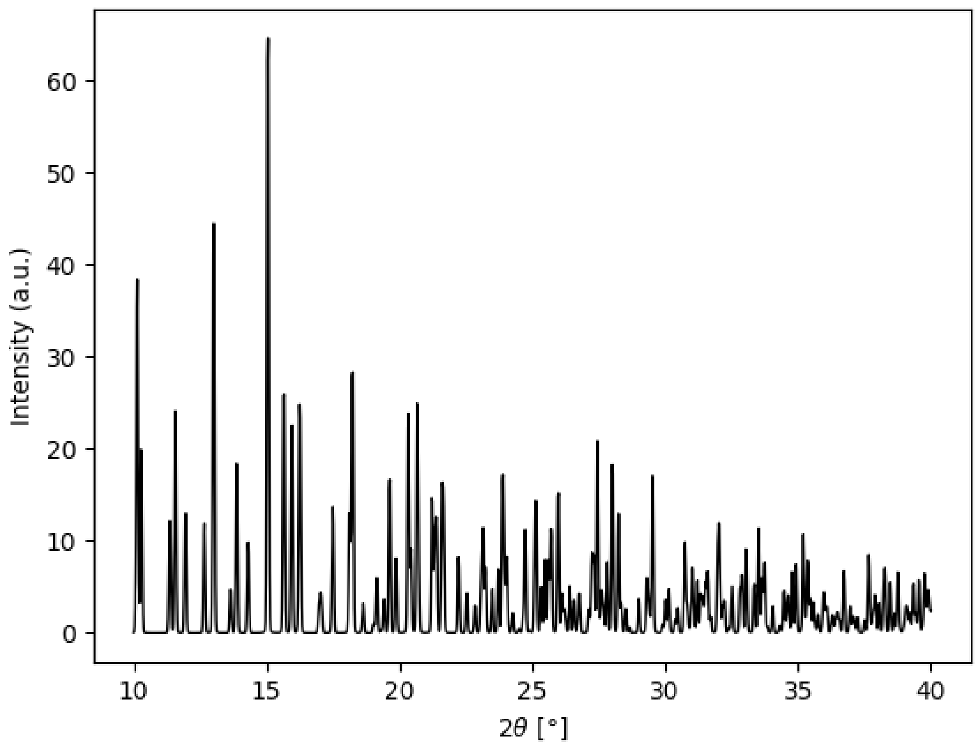

Figure S4: Simulation of powder diffraction pattern with a wavelength of 1.4 Å on the X-ray diffraction structure of **1**.

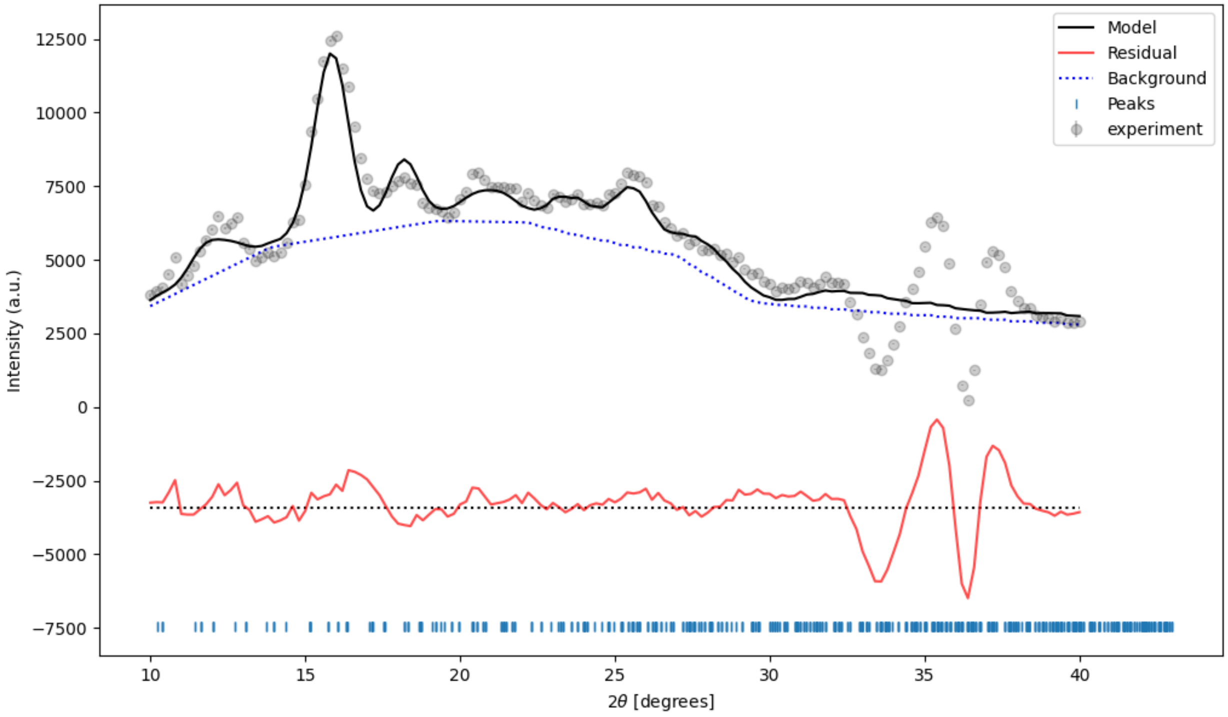

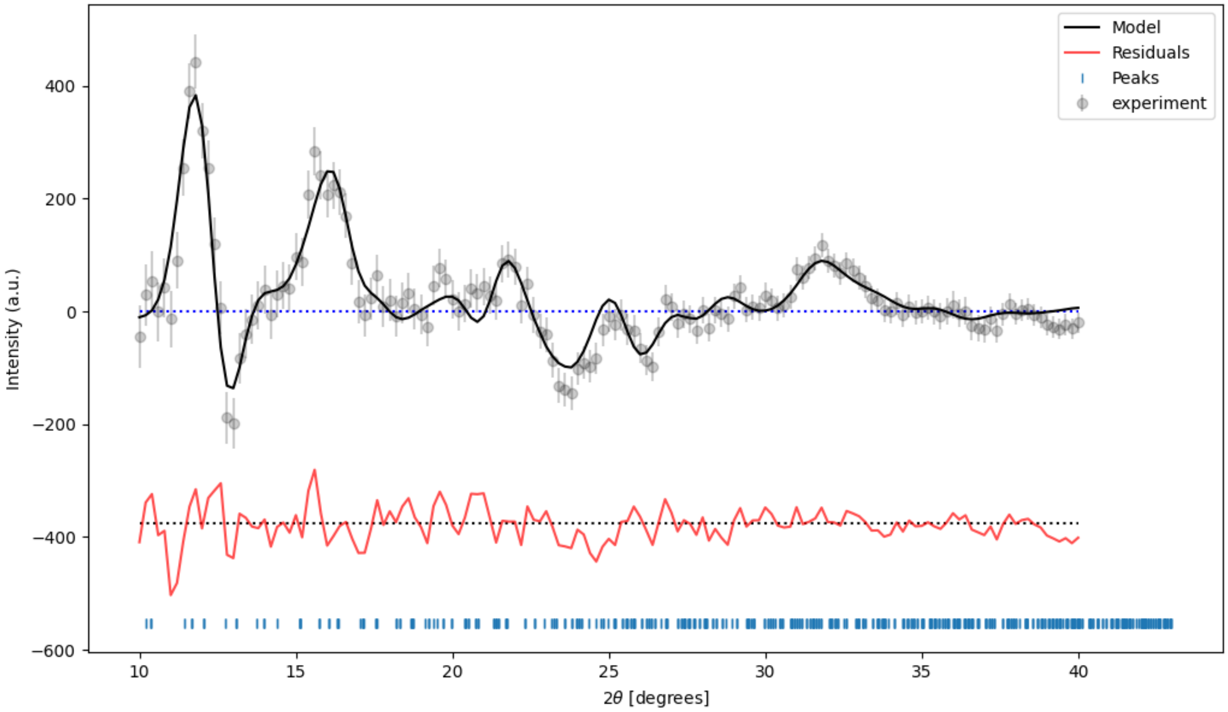

Figure S5: Projection of sum (top) and difference (below) diffraction patterns on the Bragg angle 2θ, calculated from the polarized neutron powder diffraction experiment on **1**.

The refinement does therefore not consider the crystallographic structure, but only the magnetic structure, which is seen most clearly in the difference pattern (Figure S5, bottom). This was

modelled only through the 6 independent elements of the site susceptibility tensor of Co(II) in the crystallographic structure. The sum pattern was described by the offset of the beam in 2θ and the angle of the diffraction cone base, φ, the preferred orientation (as described by the modified March's function (Kibalin & Gukasov, 2019)) a scale factor and the background, defined by a set of points and the linear interpolation between those points. We made an attempt at making a combined refinement, but the sum pattern dominated the residual function, so this did not give a good description of the magnetic structure, and as the program does currently not allow for a custom weighting between the sum and difference patterns, the susceptibility parameters were refined independently from the other parameters and only to the difference pattern. Due to the imperfect treatment of the aluminium-contribution in the sum pattern, the refinement of the sum pattern was only performed up to 30˚ in 2θ. The refinement of the difference pattern was performed up to 40˚ in 2θ, and both patterns were refined up to 40˚ in φ.

The final model gave χ^2-values (based on the pixel values in the 2-dimension diffraction pattern) of 52.7 for the sum pattern and 0.287 for the difference pattern. The full 2-dimensional modelling of the data is shown in Figure S6. We note that the axis for preferred orientation was determined to have the direction (h_{ax}, k_{ax}, l_{ax}) = (-1.773, 0.122, 7.602), which is primarily along the c*-axis of the structure. Seeing that the magnetic susceptibility tensors of the 4 molecules in the unit cell are oriented to give a unit cell susceptibility that has a strong contribution along the c-axis of the crystal structure, as shown in Figure S7, this strengthens the assertion that the preferred orientation is determined by the torque induced reorientation of crystallites in the magnetic field during the experiment. Along the same lines, the parameter describing the crystallite shape is refined to a value of $0.8643 < 1$, which in the modified March's function is a sign of platy crystallites. In other words, the refinement shows signs of a bistable directionality of the crystallites. This is conceptually the same as, an thus supports the view of, magnetically axial crystallites, as an axial anisotropy of the crystallites gives rise to a directional bistability in the magnetic field that causes the crystallite reorientation.

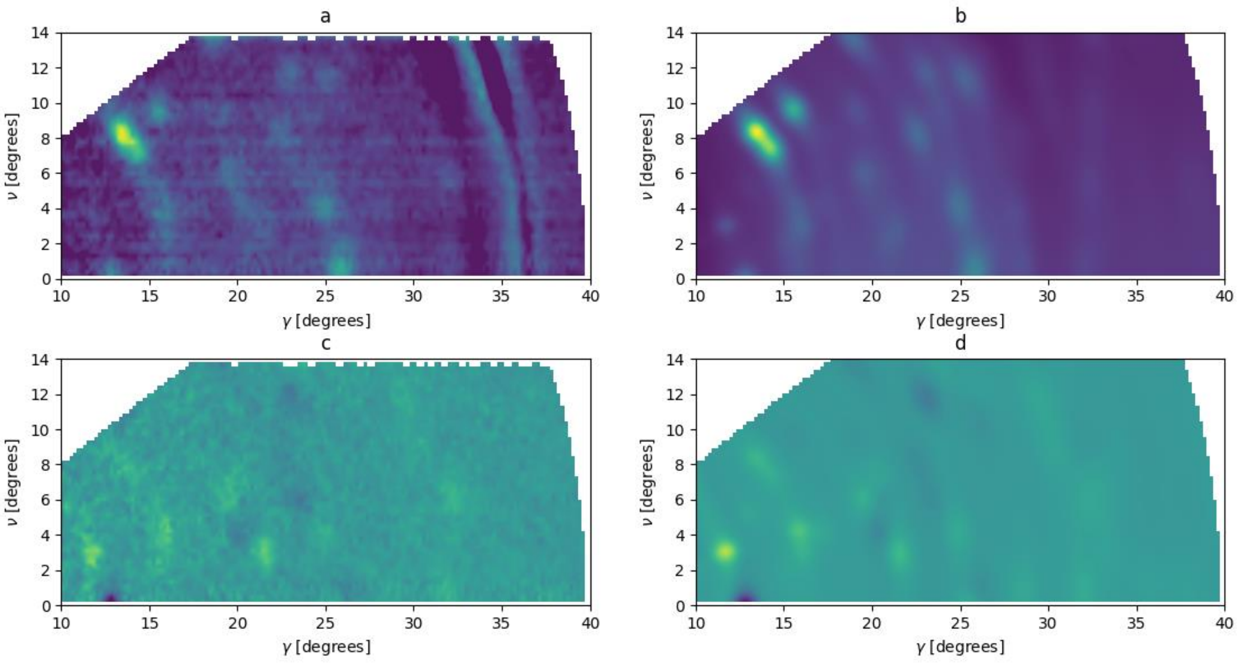

Figure S6: Modelled sum (b) and difference (d) patterns of **1** based on the sections of the experimental sum (a) and difference (c) maps that have been extracted from the full data set shown in Figure 8.

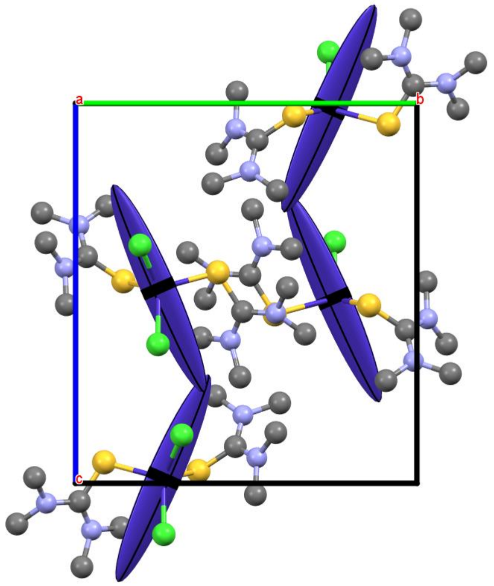

Figure S7: Orientation of the site susceptibilities of Co(II) in **1**. The summation of these 4 ellipsoids shows that the crystallite susceptibility is primarily located along the c-axis of the crystallographic unit cell. Hydrogens have been omitted from the molecular structures.

Methodological background, data description and refinement of polarized neutron diffraction on $CoBr_2(tmtu)_2$

The PND study is based on the measurement of the Bragg intensities for two different polarization states of the incident neutron beam I_+ and I_-. These intensities depend on the polarization of the incoming neutron beam, through the beam polarization vector $\boldsymbol{P} = \boldsymbol{u}P$, where P is the polarization factor of the beam, and $\boldsymbol{u}$ is a unit vector giving the direction of the beam polarization. In the experiment, $\boldsymbol{u}$ can be set either parallel ($\boldsymbol{P}_+$) or antiparallel ($\boldsymbol{P}_-$) to the vertically applied magnetic field on the sample position. The intensities are then given by

$$I_\pm \propto F_N^2 + F_{M\perp}^2 + \boldsymbol{P}_\pm \cdot (F_N^* \boldsymbol{F}_{M\perp} + F_N \boldsymbol{F}_{M\perp}^*) \tag{3}$$

where F_N and $\boldsymbol{F}_M$ are the nuclear and the magnetic structure factors, respectively, and the symbol * denotes the complex conjugate. Assuming that magnetization is localized on the atoms, the magnetic structure factor $\boldsymbol{F}_M$ can be defined as a vector sum over all the magnetic moments in the unit cell:

$$\boldsymbol{F}_M(\boldsymbol{Q}) = \sum_{a}^{\text{unit cell}} \boldsymbol{m}_a f_a(\boldsymbol{Q}) \exp(i\boldsymbol{Q} \cdot \boldsymbol{r}_a)\,, \tag{4}$$

where $f_a(\boldsymbol{Q})$ is the magnetic form factor of atom a, and $\boldsymbol{F}_{M\perp}$ refers to the component of $\boldsymbol{F}_M$ perpendicular to the scattering vector $\boldsymbol{Q}$. The central assertion of the susceptibility tensor model is that the intensities $I_\pm$ containing information on both the orientation and the magnitude of the atomic magnetic moments throughout the unit cell can be expressed in terms of a local susceptibility tensor $\overline{\chi_a}$. It is a second rank symmetric tensor describing the magnetic response of an atom to an applied magnetic field

$$\boldsymbol{m}_a = \overline{\chi}_a \boldsymbol{B}. \tag{5}$$

In the most general case, the tensor $\overline{\chi}_a$ is symmetric across the diagonal and therefore contains three independent diagonal components and three independent off-diagonal components. These can be modelled to the experimental data by using the combination of equations (3)-(5).

The single crystal PND study of the susceptibility tensor is thus based on measuring the polarization-dependent Bragg peak intensities for (at least) three orientations of a single crystal with respect to an applied magnetic field. The advantage of the polarized beam is that the so-called flipping ratio

$$R = \frac{I_+}{I_-} \tag{6}$$

increases the sensitivity to magnetic scattering, as seen in the appearance of the cross-term in equation (3). For the unpolarized beam, the flipping ratio trivially equals 1, and for a polarized

beam, a change in the flipping ratio of a Bragg-peak when the angle between an applied field and the crystal axes changes is a direct consequence of molecular magnetic anisotropy.

Data analysis of 2

The single-crystal diffraction data on **2** was collected with a wavelength of 1.4 Å on the 6T2 diffractometer operated by the Laboratorie Léon Brillouin (LLB) on the Orphée reactor.(Gukasov *et al.*, 2007) The diffraction data were collected under 5 different orientation of the magnetic field with respect to the crystal at a temperature of 3 K and a field strength of 1 T. The data for each orientation were obtained by setting the position of the position sensitive detector in the horizontal plane of the diffractometer, and for each detector setting, a full rotation of the sample was performed around the vertical axis of the diffractometer, bringing different Bragg-peaks into diffracting conditions.

The orientation matrices for each orientation were refined based on the nuclear structure of **2** and by using an in-house program, *indexus*, from the LLB. From the orientation matrices and knowledge of the orientation of the magnetic field in the laboratory frame, we calculate the individual magnetic field orientations with respect to the molecule in the asymmetric unit of **2**, which is shown in Figure S8. The flipping ratios extracted for each orientation are shown as a function of the scattering angle, 2θ in Figure S9. For orientations 3 and 5, only the data collected at a detector position of $17°$ were available, while for orientations 1, 2, and 4, data for a second detector position of $37°$ were used as well. The number of reflections obtained for the refinement from each orientation, are presented in Table S4.

The final refinement of the site susceptibility tensor for Co(II) in **2** was based on the nuclear structure determined for the compound, and presented in SI X-1, and gave a combined χ^2-value of 1.927 based on a total of 264 flipping ratios.

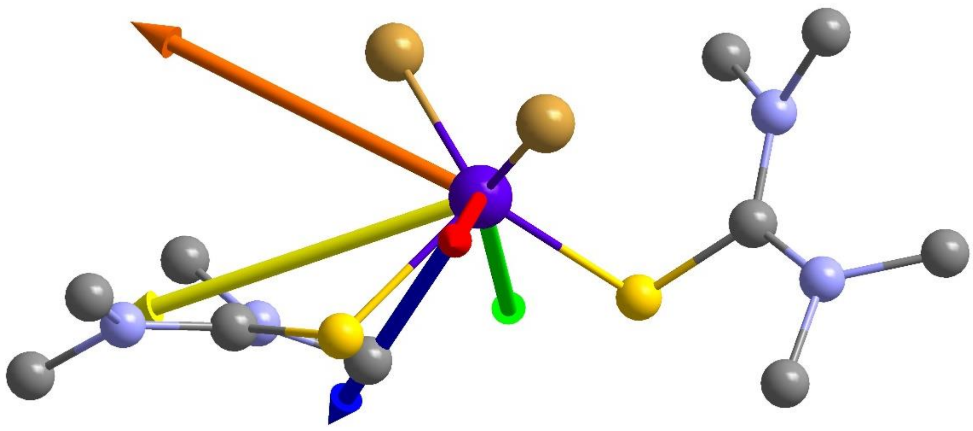

Figure S8: Molecular structure of 2 with hydrogens omitted. The red, green, blue, yellow, and orange arrows correspond to the first, second, third, fourth, and fifth orientations of the magnetic field respectively. Atom colors are purple, brown, yellow, blue, and gray for Co, Br, S, N, and C.

Table S4. Details on the refinement of single-crystal polarized neutron diffraction data on **2**. Columns correspond to the different orientations of the crystal.

	**1**	**2**	**3**	**4**	**5**
N_{meas}	75	34	21	102	32
χ^2	1.478	1.221	1.347	1.418	5.730

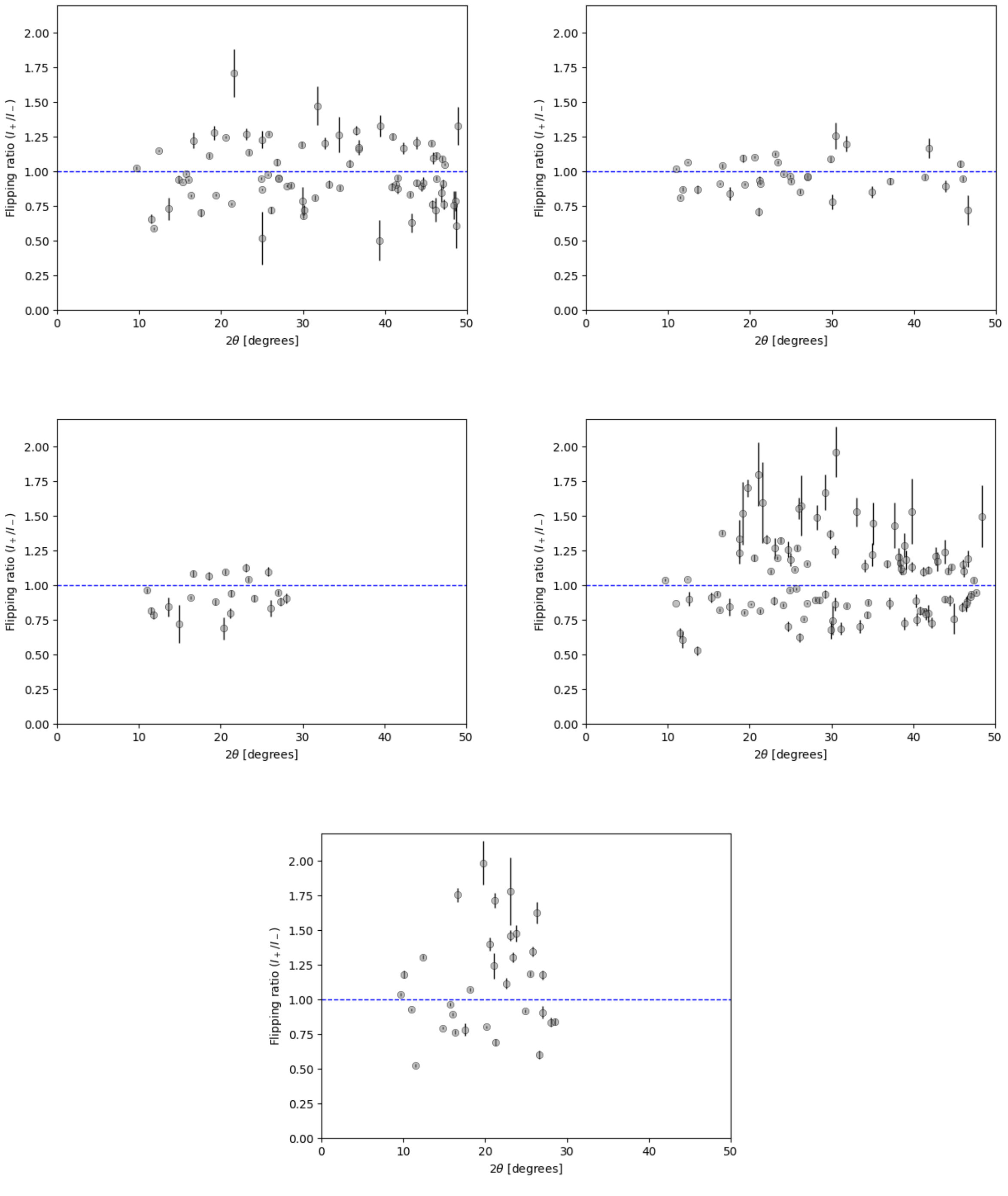

Figure S9: Flipping ratio data for **2** collected for the five different orientations with orientation 1, 2, 3, 4 and 5 appearing from top to bottom, left to right. The blue, horizontal line at a flipping ratio of 1 corresponds to

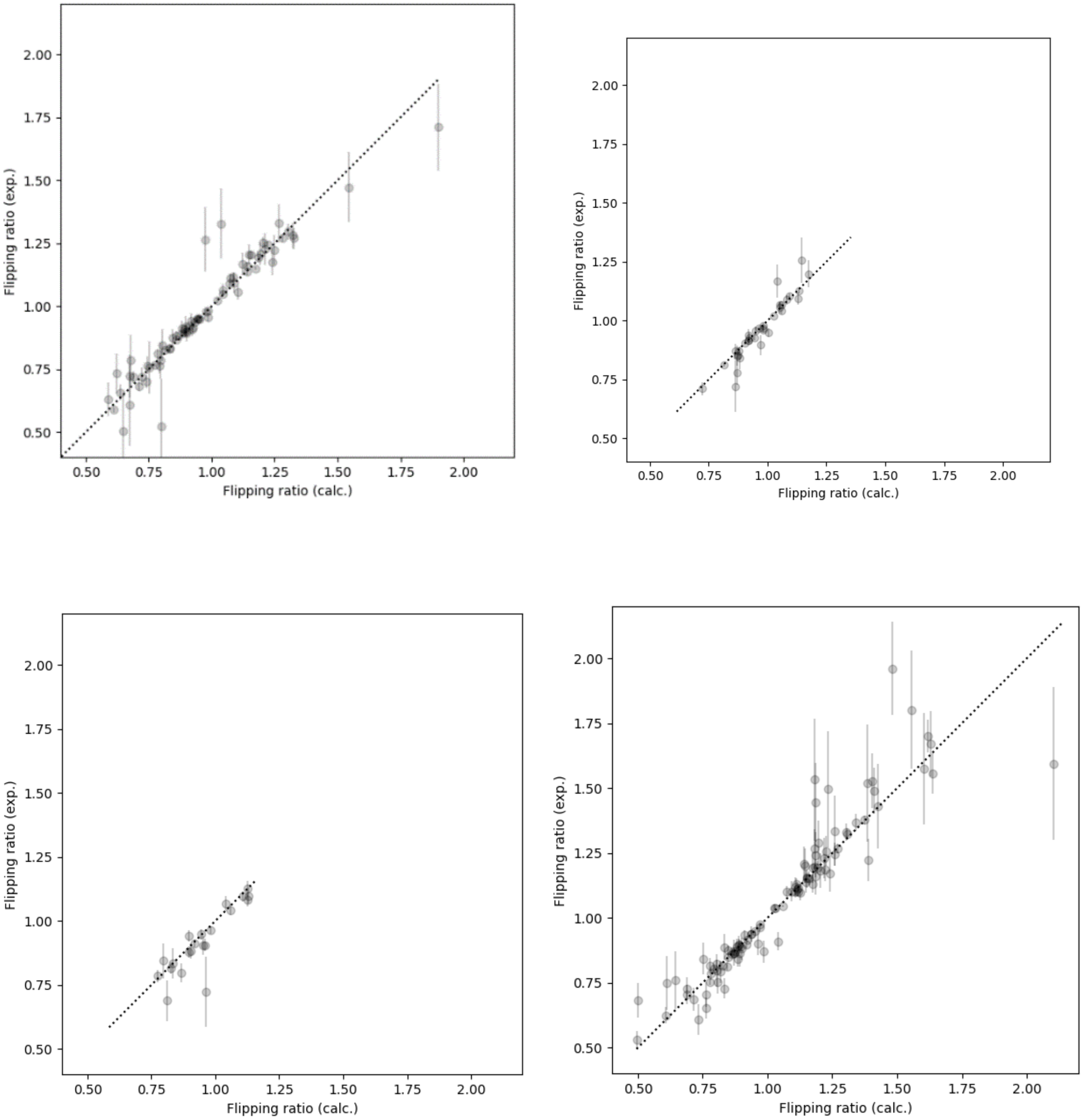
Flipping ratio (exp.)
Flipping ratio (calc.)
0.50
0.75
1.00
1.25
1.50
1.75
2.00

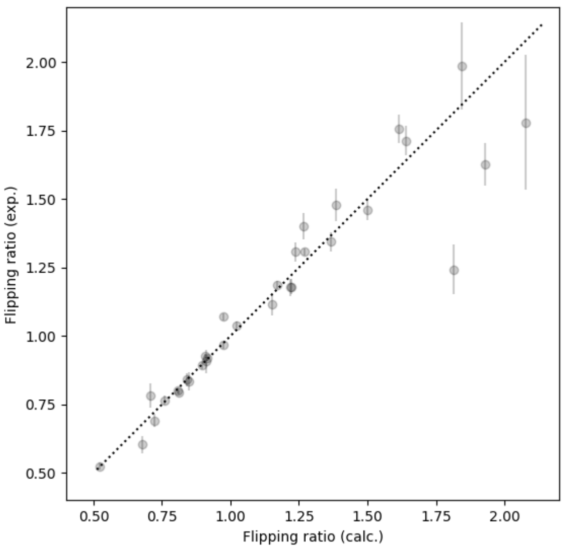

Figure S10: Calculated vs. experimental flipping ratios for the 5 orientations (1 to 5, top to bottom) that were measured. The dotted diagonal line corresponds to perfect agreement between model and experiment.

REFERENCES

Clark, T., Hennemann, M., Murray, J. S. & Politzer, P. (2007). *J. Mol. Model.* **13**, 291-296.

Gukasov, A., Goujon, A., Meuriot, J.-L., Person, C., Exil, G. & Koskas, G. (2007). *Physica B: Condensed Matter* **397**, 131-134.

Kibalin, I. A. & Gukasov, A. (2019). *Phys. Rev. Research* **1**, 033100.

Politzer, P. & Murray, J. S. (2019). *Crystals* **9**, 165.

Vaidya, S., Shukla, P., Tripathi, S., Rivière, E., Mallah, T., Rajaraman, G. & Shanmugam, M. (2018). *Inorg. Chem.* **57**, 3371-3386.
